# Epigenetic mechanisms of Strip2 in differentiation of pluripotent stem cells

**DOI:** 10.1038/s41420-022-01237-5

**Published:** 2022-11-05

**Authors:** Sureshkumar Perumal Srinivasan, Harshal Nemade, Anna Cherianidou, Luying Peng, Sara Cruz-Molina, Alvaro Rada-Iglesias, Agapios Sachinidis

**Affiliations:** 1 Faculty of Medicine and University Hospital Cologne, Center for Physiology, Working Group Sachinidis, University of Cologne, 50931 Cologne, Germany; 2 Heart Health Center, Shanghai East Hospital, School of Medicine, Tongji University, 200120 Shanghai, China; 3 Research Units of Origin and Regulation of Heart Rhythm, Chinese Academy of Medical Sciences, 100730 Beijing, China; 4 Department of Medical Genetics, Tongji University School of Medicine, 200092 Shanghai, China; 5 Max Planck Institute for Molecular Biomedicine, Muenster, Röntgenstraße 20, 48149 Münster, Germany; 6 Institute of Biomedicine and Biotechnology of Cantabria (IBBTEC), CSIC/University of Cantabria, Santander, Spain; 7 Center for Molecular Medicine Cologne (CMMC), University of Cologne, 50931 Cologne, Germany

**Keywords:** Gene silencing, Embryonic stem cells

## Abstract

Significant evidence points to Strip2 being a key regulator of the differentiation processes of pluripotent embryonic stem cells. However, Strip2 mediated epigenetic regulation of embryonic differentiation and development is quite unknown. Here, we identified several interaction partners of Strip2, importantly the co-repressor molecular protein complex nucleosome remodeling deacetylase/Tripartite motif-containing 28/Histone deacetylases/Histone-lysine N-methyltransferase SETDB1 (NuRD/TRIM28/HDACs/SETDB1) histone methyltransferase, which is primarily involved in regulation of the pluripotency of embryonic stem cells and its differentiation. The complex is normally activated by binding of Krueppel-associated box zinc-finger proteins (KRAB-ZFPs) to specific DNA motifs, causing methylation of H3 to Lysin-9 residues (H3K9). Our data showed that Strip2 binds to a DNA motif (20 base pairs), like the KRAB-ZFPs. We establish that Strip2 is an epigenetic regulator of pluripotency and differentiation by modulating DNA KRAB-ZFPs as well as the NuRD/TRIM28/HDACs/SETDB1 histone methyltransferase complex.

## Introduction

Embryonic stem cells (ESCs) offer the exciting opportunity to identify genes that are required for differentiation and development of somatic cells. A series of differentiation and cleavage events occur during early embryonic development, leading to the formation of distinct cell types that later form the organism. For organismal development, the transmission of selected epigenetic information from the germline to the soma is critical. This epigenetic memory is preserved in germ cell epigenomes, which later decide the totipotent embryonic state. The processes underlying this reprogramming resistance are poorly understood.

To investigate this further, the function and intracellular signaling pathways of the striatin-interacting phosphatase and kinase (STRIPAK) complex in regulating biological processes in several organisms have recently attracted attention. Strip2 is a component of the STRIPAK complex, which regulates cell growth, proliferation, migration, and adhesion, as well as brain and vascular development and heart function [1, 2]. In many eukaryotes, the knowledge of the function and structure of the STRIPAK complex-associated Strip2 protein is very poor. Strip2 has 61% amino acid identity and two distinct domains: the N-terminal N1221-like domain and the C-terminal DUF3402 domain (domain of unknown function 3402). Both domains are functionally unknown and have no resemblance to any other protein domains.

We previously demonstrated that Strip2 is required for the initiation of ESC differentiation. According to our findings, Strip2 is distributed in the perinucleus or nuclei of wild-type undifferentiated ESCs but is localized in high-density nuclear bodies in differentiated cells [3, 4]. Although Strip2-silenced murine ESCs (Strip2^−^-ESCs) were differentiated into embryoid bodies (EBs) for 16 days (16-day Strip2^−^-EBs), they still showed a strong tumorigenic potential and high expression levels of epigenetic regulator genes Hat1 and Dnmt3 [3]. In 16-day Strip2^−^-EBs, enzymatic activity of histone acetyltransferase type B (Hat1) and DNA (cytosine-5)-methyltransferase 3 beta (Dnmt3b) were higher than in control Strip2^+^-EBs. To date, the involvement of Strip2 in epigenetic regulation of stem cell differentiation is unclear.

The epigenetic regulation of developmental and differentiation processes by maintaining the process of DNA methylation requires DNA methyltransferase DNMT1, which contributes to maintaining global demethylation [5–7]. Selected regions were specifically targeted by DNMT1, which led to embryo preimplantation. For instance, the tripartite motif-containing protein 28 (TRIM28) recruits DNMT1 directly or indirectly to directed specific genomic loci by the DNA-binding protein ZFP57 [8, 9]. ZFP57 is a Krueppel-associated box (KRAB) domain zinc-finger protein (ZFP). In the mouse genome, KRAB-ZFPs are the largest subtype of C2H2-type zinc-finger transcription factors. Their KRAB domain recruits TRIM28, histone methyltransferase SETDB1, the nucleosome remodeling and histone deacetylase (NuRD) complex, and DNA methyltransferases 3 A, 3B and 1 by sequence-specific binding through their zinc-finger domains [9–12]. Through interactions with murine ZFP809, TRIM28 has been demonstrated to promote suppression of endogenous retroelements in ESCs [13]. During early embryogenesis, TRIM28s associate with ZFP57 to maintain monoallelic DNA and H3K9 methylation patterns of imprinted genes [8, 9]. KRAB-ZFPs have long been associated with transposable element silencing; their sequence-specificity is attributable to unique zinc-finger combinations that target diverse retrotransposon families [14] and leads to the recruitment of the KAP1 co-repressor complex. This stimulates the local deposition and maintenance of H3K9me3 and mediates transcriptional repression [12].

In the present study, we investigated the role of epigenetic mechanisms, in particular the role of the NuRD/TRIM28/HDACs/SETDB1 histone methyltransferase complex in the regulation of the differentiation processes mediated by Strip2. Using ChIP-Seq, we mapped putative DNA binding sites of Strip2, and with aid of Co-Immunoprecipitation (Co-IP), several protein binding partners of Strip2 involved in ESC differentiation were identified and confirmed using proximity ligation assay (PLA). In addition, we measured the H3K9 methyltransferase activity in undifferentiated and differentiated states of ESCs. Finally, we validated the impact of the NuRD/TRIM28/HDACs/SETDB1 histone methyltransferase complex by knockdown of strip2 in ESC differentiation.

## Results

### Unbiased genome-wide mapping of Strip2-DNA binding regions in mouse ESCs

**Fig. 1:**
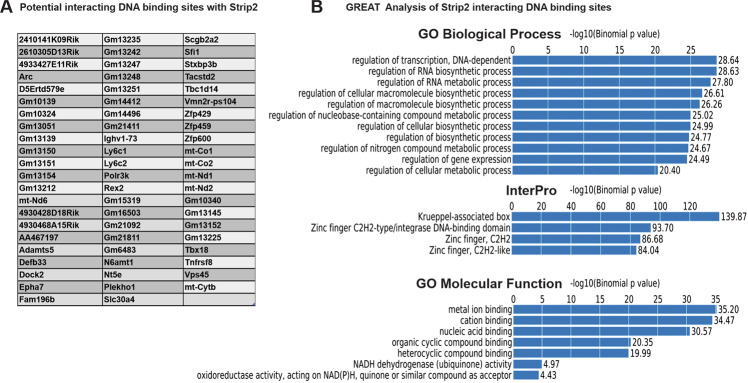
DNA motif associated with Strip2. **A** List of active binding sites of strip2 according to mm10 coordinates. **B** Top Gene Ontology Terms Significantly Enriched in Strip2 active binding sites according to Genomic Regions Enrichment of Annotations Tool (GREAT) analysis. Interestingly, the InterPro analysis indicated that Strip2 interacts with the DNA regions of several Krueppel-associated box (KRAB) zinc-finger proteins (ZFPs) which are known repressors of development.

Recently, we showed that Strip2 was localized in the nucleus of ESCs and regulates different lineage differentiations [3, 4]. Our previous transcriptome and enzymatic activity studies pointed to a potential epigenetic mechanism behind the regulatory activity of Strip2. To explore the Strip2 gene and epigenetic regulatory activity, we extended our study by performing ChIP-Seq investigations (Fig. S1). Given the lack of a ChIP validated antibody for Strip2, we used the AM-tag ChIP methodology. For this purpose, we expressed pAM-1C-Strip2 in mouse ESCs (mESCs) and performed ChIP-Seq from undifferentiated ESCs. The expression profile of genes associated with differentiation in pAM-1C-Strip2 mESCs and control mESCs were analyzed and showed no significant differences between each other (Fig. S4). ChIP-Seq maps for Strip2 in mESCs identified a total of 130 enriched regions. Using Genomic Regions Enrichment of Annotations Tool (GREAT), these regions were associated with 65 genes (Fig. 1Α). GREAT analysis indicated that pAM-1C-Strip2 ChIP-Seq peak-associated genes were related to regulation of transcription, nucleic acid binding, regulation of gene expression and the KRAB and ZFP C2H2-type/integrase DNA-binding domain (Fig. 1B), which supports our previous findings based on transcriptomic data for Strip2^−^-ESCs (Fig. 3A) [3].

**Fig. 2:**
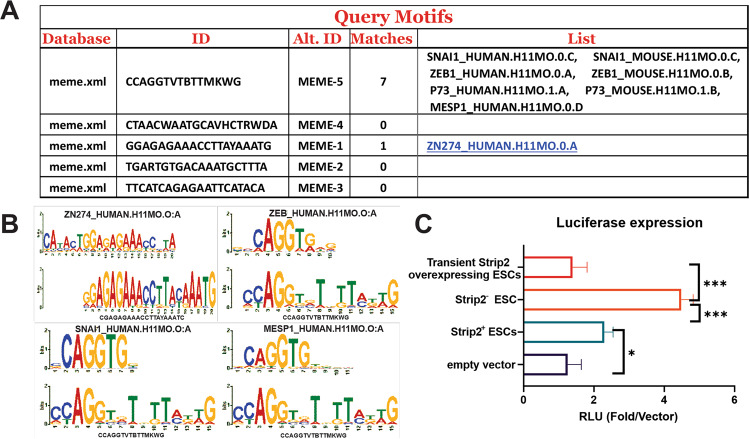
Top motif hits of DNA motif associated with Strip2 identified via SeqPos motif tool analysis. **A** The five best hits are shown. Out of five motifs, motif 1 and 5 have matches to ZNF274 and SNAI1, EXB1 and MESP1. **B** Motifs significantly enriched in Strip2 mESCs were identified by analysis of motif enrichment (AME). **C** Luciferase expression in Strip2 ^+^ -ESCs show that the absence of Strip2 (Strip2 ^−^ -ESCs) further stimulates expression, while Transient overexpression of Strip2 reduces expression to baseline levels. Average fold change of relative luciferase units (RLU) vs empty vector (*n* = 3, mean ± SD).

**Fig. 3:**
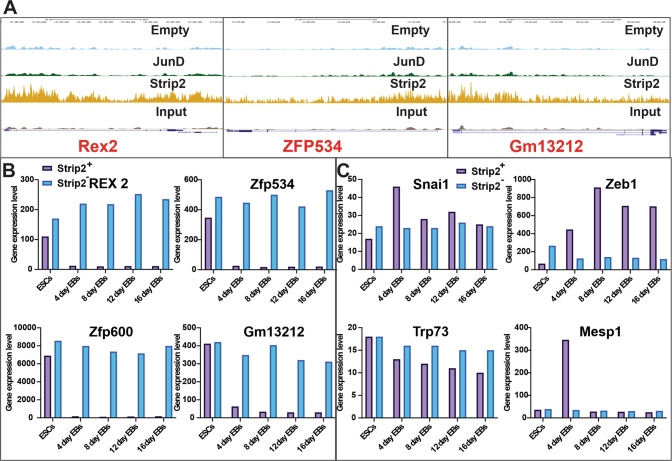
Strip2 ChIP-Seq profiles of Rex2, Zfp534 and Gm13212 genes, their expression and the Strip2 motif targeted genes expression pattern during differentiation of mESCs. **A** Strip2 ChIP-Seq profiles generated in mouse ESCs of Rex2, ZFP534 and Gm13212 with control (empty vector and JunD). **B** Strip2 ChIP-enriched gene expression pattern in transcriptomic data of ESCs differentiation from day 0 to day 16. **C** Motif targeted gene expression pattern in transcriptomic data of ESCs differentiation from day 0 to day 16. ChIP-Seq: Chromatin Immuno-Preciptiation Sequencing.

These ChIP-Seq data were analyzed using the MEME analysis tool to find the DNA motif of Strip2. The motif analysis revealed that the KRAB-ZFP motif –MEME-1-GGAGAGAAACCTTAYAAATG-was the most significant motif (Y stands for not clearly identified nucleotide). BLAST analysis using the above motifs (20 bp) also was conducted (https://blast.ncbi.nlm.nih.gov/Blast.cgi). The BLAST analysis identified several mouse ZFPs and predictive ZFP-like genes (Fig. 2A, B). Along with *Snal1* and *Zeb1* (KRAB-ZFP motif), we also discovered binding sites for *Rex2*, *Zfp534* and *Gm13212* (Fig. 3B, C) from our ChIP-Seq data. Notably, this analysis helped confirm the presence of the KRAB-ZFPs and their associated proteins. To independently confirm the DNA motif associated with Strip2 (GGAGAGAAACCTTAYAAATG) identified by ChIP-Seq, we performed luciferase reporter assays (Fig. 2C). In the Strip2 ^−^ -ESCs, the pGL3 minimal promoter had comparatively higher levels of luciferase expression, suggesting that the DNA binding motif discovered by our ChIP-Seq is highly specific to Strip2. In contrast, the Strip2 overexpressed ESCs had significantly lower levels of luciferase expression, when compared to the Strip2^−^-ESCs, validating the specificity of DNA motif associated with Strip2 in the mouse genome (Fig. 2C). In addition, we confirmed the enriched gene from our ChIP-Seq data with our microarray data and validated by qPCR, which clearly indicated that KRAB-related enriched genes were highly upregulated in Strip2^−^-ESCs, when compared to the control scrambled Strip2^+^-ESCs (Fig. 3B, C and Fig. S3). In conclusion, chromosomal distribution patterns of Strip2 clearly participate in the transcriptional repression pathway via KRAB-ZFPs.

### Identification of Strip2 interaction partners in mESC differentiation

**Fig. 4:**
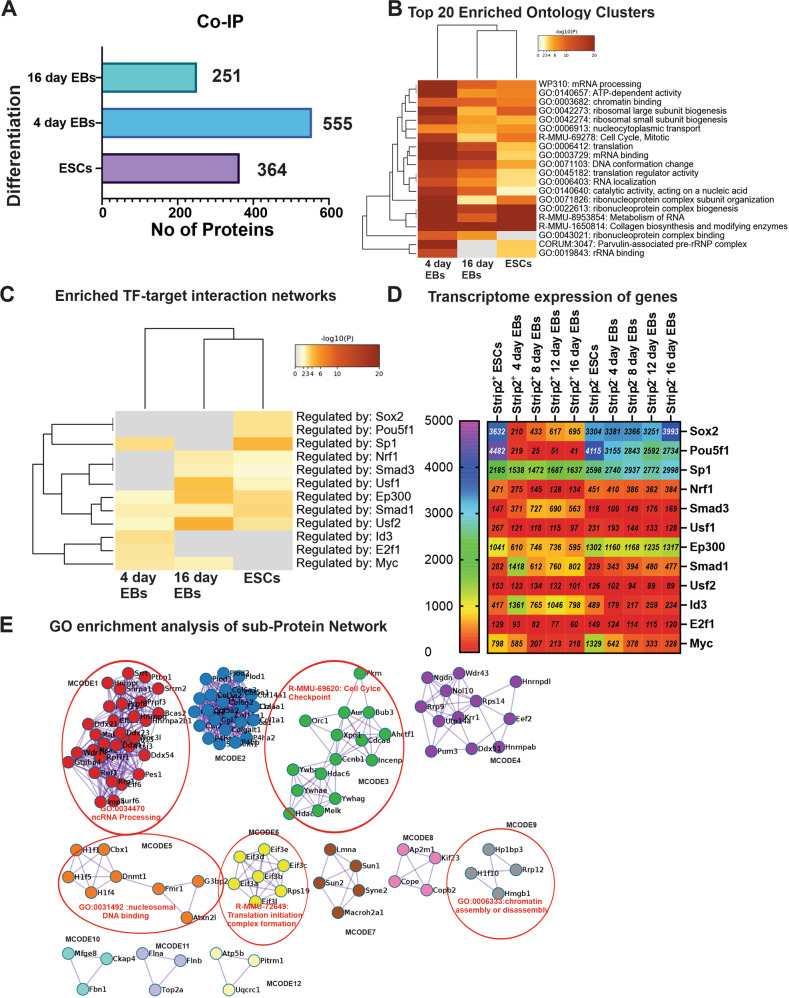
Proteins interacting with Strip2 during murine ESC differentiation. **A** Bar graph representing number of proteins that were interacting with Strip2 at different points of differentiation (data derived using immunoprecipitation method). **B** Visualization of enriched ontology terms of proteins interacting with Strip2 in ESCs, 4-day and 16-day EBs. Heatmap showing the top 20 enrichment clusters, colored by *p* values. **C** Metascape enrichment analysis of all statistically enriched transcription factor-target interaction networks. **D** Gene expression profile of enriched transcription factor-target interaction networks. **E** GO enrichment analysis was applied to each MCODE network to assign “meanings” to the network component, where the top three best *p* value terms were retained. MCODE components were identified from the merged network. Each MCODE network is assigned a unique color.

Our ChIP-Seq results suggested that Strip2 is vital to the transcriptional repression pathway and epigenetic regulation. To unravel Strip2 molecular partners involved in pluripotency and epigenetic regulation, we performed Co-IP by generating an ESC cell line overexpressing Strip2-turboGFP in undifferentiated ESCs (Fig. S2). In parallel, a control ESC cell line was generated using a control plasmid without the Strip2. The Co-IP analysis identified 364 partners in 0-day ESCs, 555 partners in 4-day EBs and 251 partners in 16-day EBs (Fig. 4A, Table S1). To investigate how, and with which molecular partners, Strip2 regulates different biological processes, we involved all molecular partners obtained at each time point (0-day ESCs, 4-day EBs and 16-day EBs) in the enriched ontology clusters and protein-protein interaction network analysis using the Metascape tool (Fig. 4B). The heatmap revealed a lot of functional overlap (*P* < 0.05, false discovery rate (FDR)-corrected) between 0-day ESCs, 4-day EBs and 16-day EBs, although these proteins were probably involved in different parts of the same epigenetic and developmental processes, related to most significantly enriched ones, such as ‘GO:0003682: chromatin binding’, ‘WP310: mRNA processing’ and ‘GO:0006913: nucleocytoplasmic transport’. Moreover, ‘R-HSA-69278: Cell Cycle, Mitotic’ and the ‘R-HSA-212165 and R-MMU-2995410: Nuclear Envelope (NE) Reassembly’ were also enriched (Reactome database). Notably, the ‘GO:0019827: stem cell population maintenance in 0-day ESCs and 4-day EBs were enriched and ‘GO:0031570: DNA integrity checkpoint signaling’ were enriched at day 0 (Table S2). We also identified significant enrichment transcription factor motifs of Sox2 and Pou5f1 at ESCs, Id3 and Smad1 at day 4; and Ep300 and Usf2 in Strip2 interacting protein network at day 16 (Fig. 4C, Table S3). The gene expression profile of aforementioned transcription factors from our ESCs Strip2^−^-ESCs is shown in Fig. 4D.

Moreover, to understand the protein subnetworks involved in each of these significant biological processes, we performed analysis using MCODE, an algorithm accessed via the Metascape tool (Fig. 4E). The analysis identified twelve protein interaction subnetworks (Fig. 4E). Each of these subnetworks regulate a key biological process, such as ‘GO:0034470: ncRNA Processing’, ‘R-MMU-69620: Cell cycle Checkpoint’, ‘GO:0031492: nucleosome DNA binding’, ‘R-MMU-72649: Translation initiation complex formation’, and ‘GO:0006333: chromatin assembly or disassembly’ (see Table S4). These results demonstrated that the Strip2 mainly interacts with nucleus/nucleolar proteins, which are associated with ribosomal biogenesis and RNA splicing processes. In summary, our findings suggest that Strip2 regulates gene expression by interacting on an epigenetic level with several nuclear molecular entities.

### Strip2 regulates gene expression by interacting with TRIM28 and KRAB-ZFPs

**Fig. 5:**
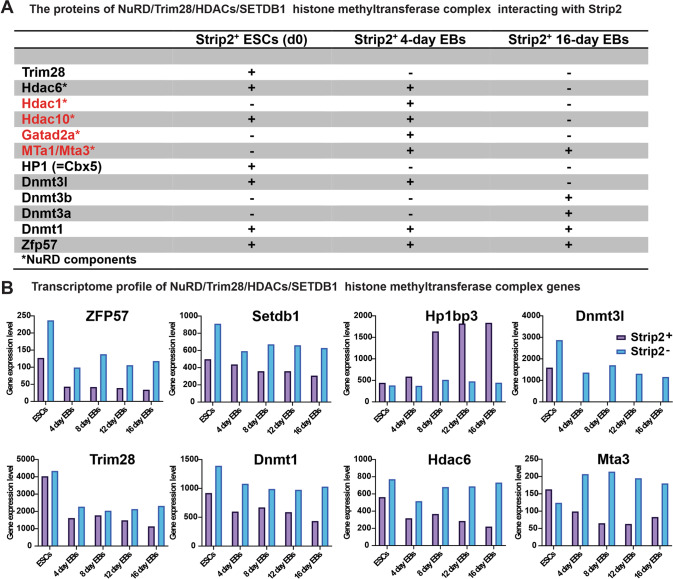
Transcriptional repression complex: Proteins interacting with Strip2. **A** List of proteins from nucleosome remodeling and histone deacetylase (NuRD) components interacting with Strip2 during different points of differentiation from day 0 to day 16. **B** Gene expression profile of transcriptional repression complex genes involved epigenetic regulation.

**Fig. 6:**
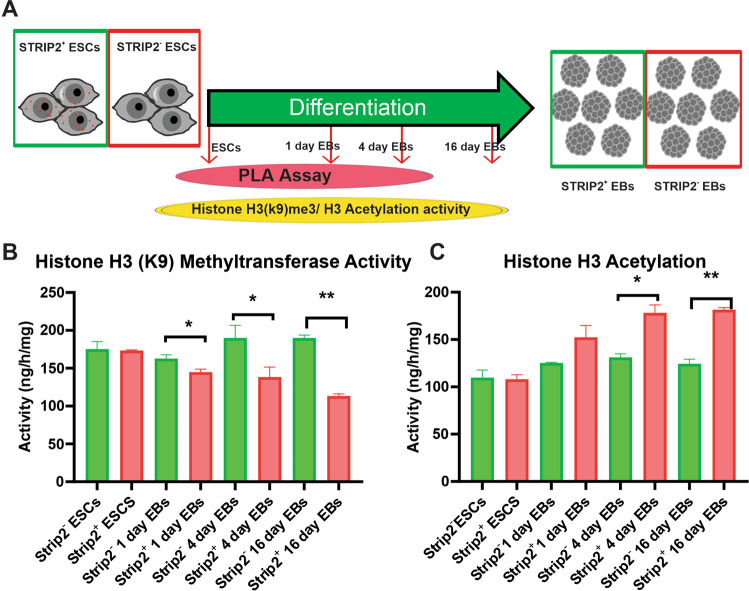
Proximity ligation assay and enzyme assay results. **A** Schematic workflow represents the analysis of proximity ligation assay (PLA) and enzyme assay at different points of differentiation between Strip2^−^-ESCs and Strip2^+^-ESCs. **B** Quantification of H3K9 methyltransferase activity in undifferentiated and differentiated Strip2^−^-ESCs and control Strip2^+^-ESCs, respectively (*n* = 3). Mean ± SEM values are shown. **C** Histone H3 acetylation activity in undifferentiated and differentiated Strip2^−^-ESCs and control Strip2^+^-ESCs, respectively (Mean ± SEM values, *n* = 3). SEM: Standard error of the mean.

**Fig. 7:**
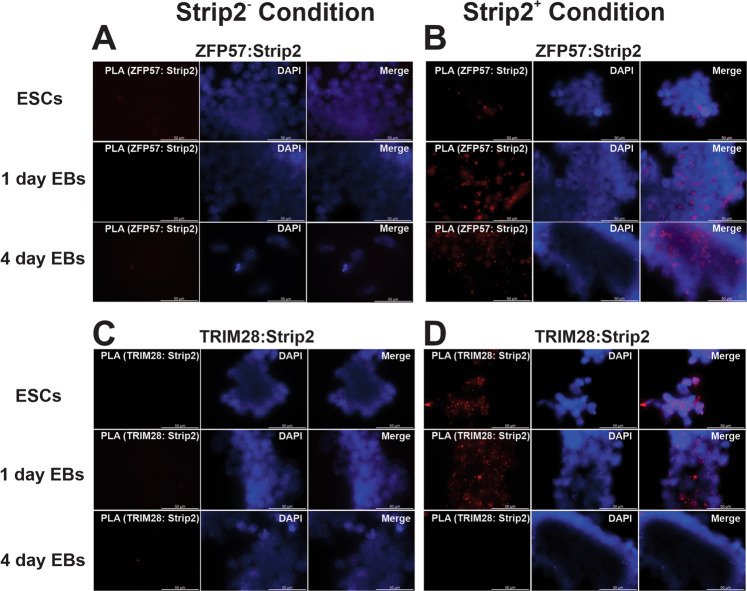
Representative images from proximity ligation assay (PLA): Assays were performed on Strip2^−^-ESCs vs Strip2^+^-ESCs at different time point of differentiation (0-day, 1-day and 4-day EBs). **A** Absence of proximity (distance < 40 nm) red dots between Strip2 and ZFP57 in Strip2^−^-ESCs condition in all three time points. **B** Evidence of proximity (distance < 40 nm) red dots found between Strip2 and ZFP57 in all three time points. **C** Absence of proximity (distance < 40 nm) red dots between Strip2 and TRIM28 in Strip2^−^-ESCs condition in all three time points. **D** Strip2 and TRIM28 proximity (distance < 40 nm) red dots were found only in 0-day ESCs and 1-day EBs but not in 4-day EBs. Nuclei are counterstained with DAPI (blue fluorescent stain). Scale bar: 50 μm.

To explore how Strip2-DNA binding to the KRAB-ZFP motif regulates pluripotency and differentiation in ESCs, we looked for the molecular complexes previously reported to regulate ESC pluripotency in the Strip2 Co-IP data. Strikingly, NuRD/TRIM28/HDACs/SETDB1 histone methyltransferase complex proteins are among the several partners of Strip2 (Fig. 5A). To identify the expression profile of the histone methyltransferase complex, we analyzed our Strip2^−^-ESCs transcriptome data. The loss of Strip2 resulted in the increased expression of *Dnmt3I*, *Mta3*, and *Hdac6* in 16-day Strip2^−^-EBs, compared to the control 16-day Strip2^+^-EBs (Fig. 5B). To validate NuRD/TRIM28/HDACs/SETDB1 histone methyltransferase complex interaction with Strip2, we did PLAs at different time points of differentiation (Figs. 6A, 7). During the initial stages of the control ESC differentiation at day 0, day 1 and day 4, we found that direct interaction of Strip2 with ZFP57 was substantiated by the PLA (Fig. 7A, B). In contrast, the direct interaction of Strip2 with TRIM28 was found only in 0-day ESCs and 1-day EBs, but not in 4-day EBs (Fig. 7C, D). As a negative control, we performed PLAs in Strip2^-^-ESCs. These finding confirmed that Strip2 is involved in epigenetic silencing via TRIM28 and KRAB-ZFPs.

We hypothesized from our Stable Isotope Labeling by/with Amino acids in Cell culture (SILAC) and Co-IP analyses that histone modifications, such as methylation and acetylation, are the molecular mechanisms involved in the Strip2-mediated regulation of ESC differentiation. Two enzymes, histone H3 (K9) methyl transferase and histone H3 acetylation, are involved in ESC differentiation. To evaluate our hypothesis, we analyzed H3 (K9) methyl transferase and histone H3 acetylation activity in Strip2^−^-ESCs and Strip2^+^-ESCs, as well as in their 4-day and 16-day EBs (Fig. 6B, C). During the initiation of differentiation, there was a decrease in the activity of histone H3 (K9) methyl transferase to initiate the transcription process in Strip2^+^-ESCs, while H3 (K9) methylation occurring in the Strip2^+^-ESCs significantly decreased (Fig. 6B). In contrast, the histone H3 (K9) methyl transferase activity remained high in 4-day and 16-day Strip2^−^-EBs, being significantly higher than in 4-day and 16-day Strip2^+^-EBs. Meanwhile, H3 acetylation was higher in 4-day and 16-day EBs but had significantly lower activities in Strip2^−^-EBs, when compared with Strip2^+^-EBs (Fig. 6C). These findings suggest that the Strip2 interacting proteins, such as Hadc1, Hp1, and Dnmt3b, correlate well with the increased activity of histone H3 (K9) methyl transferase and histone H3 acetylation.

Based on our results, we propose that Strip2 triggers an additional regulatory mechanism to the existing gene regulatory network, whereby regulating pluripotency and differentiation in ESCs (Fig. 3B, C). However, despite knowing that Strip2 is associated with KRAB-ZFPs, we still do not know whether Strip2 binds directly to regulatory elements, like the KRAB-ZFPs, or indirectly through proteins interacting with the KRAB-ZFP complex, thereby inactivating the effects of ZFPs.

### Impact of Strip2 knockdown on the global proteome of differentiated mESCs

**Fig. 8:**
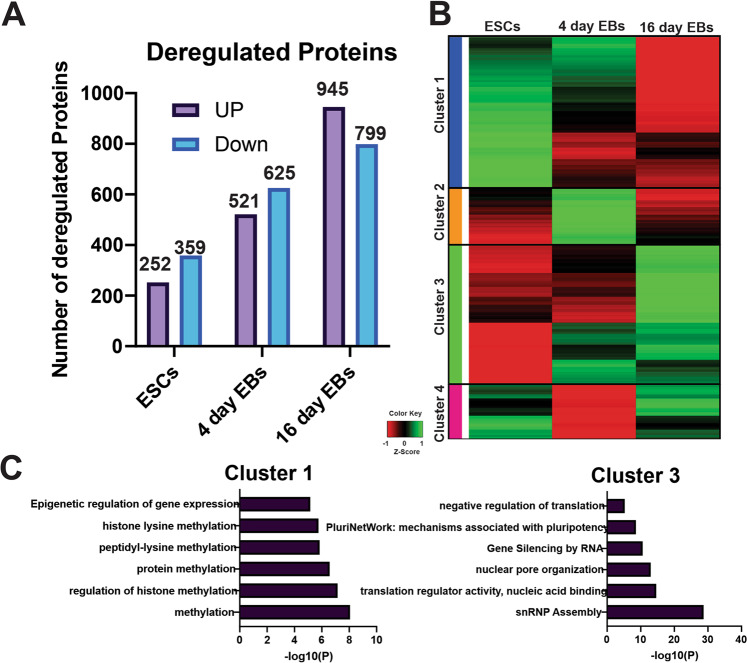
Global deregulated proteins in Strip2 KD ESCs (SILAC). **A** Number of proteins up- or downregulated in Strip2^−^-ESCs vs Strip2^+^-ESCs, 4-day Strip2^-^-EBs vs 4-day Strip2^+^-EBs, and 16-day Strip2^−^-EBs vs 16-day Strip2^+^-EBs. **B** Visualization of K-means clustering of 3 501 differentially expressed proteins (0.5-fold change in expression), using a Euclidean distance measurement and k = 4 group clusters. The replicates are displayed on the vertical axis, with proteins on the horizontal axis. *Z* score signal intensities are depicted, using a color gradient. The heatmap indicates high expression levels in red, intermediate in dark black, and low expression levels in green. **C** Metascape analysis of the deregulated proteins in clusters 1 and 3 (Strip2^−^-ESCs, 4-day EBs and 16-day EBs vs Strip2^+^-ESCs, 4-day Strip2^+^-EBs and 16-day Strip2^+^-EBs).

Quantitative global proteome analysis at various stages of differentiation of the Strip2^−^-ESCs and Strip2^+^-ESCs (0-day ESCs, 4-day and 16-day EBs) was performed using SILAC technology. A comparison of the proteomes is shown in Fig. 8A. After statistical analysis (fold change of 0.5 in both upregulated and downregulated proteins) and FDR correction (*P* < 0.05, Table S5), the K-means clustering of 3501 deregulated proteins was performed to identity various cluster patterns in the global proteome at each time point due to loss of Strip2 in mESCs (Fig. 8B). The K-means clustering indicated a differential pattern in the protein expression and segregated into four different clusters (Cluster1, Cluster2, Cluster3 and Cluster4; Table S6), based on the expression profiles. We analyzed each time point in detail to understand temporal kinetics of this protein expression. The comparison between undifferentiated (0-day) Strip2^−^-ESCs vs undifferentiated (0-day) Strip2^+^-ESCs indicated 252 upregulated (*P* < 0.05) and 359 downregulated proteins. A comparison of the 4-day Strip2^−^ vs 4-day Strip2^+^-EBs identified 521 upregulated and 625 downregulated proteins. Finally, comparison of the 16-day Strip2^−^vs 16-day Strip2^+^-EBs resulted in 945 and 799 upregulated and downregulated proteins, respectively (Fig. 8A). To understand the biological significance of the deregulated proteins, we performed a Metascape analysis (*P* < 0.05, Fig. 8C) on two specific clusters obtained from K-means clustering. Interestingly, the analysis revealed that various molecular functions, signaling pathways, and metabolic pathways were affected due to loss of Strip2. In particular, ‘GO:0032259: methylation’, ‘R-MMU-212165: Epigenetic regulation of gene expression’, ‘R-MMU-211000: Gene Silencing by RNA’, ‘WP1763: PluriNetWork: mechanisms associated with pluripotency’, ‘GO:0017148: negative regulation of translation’, and ‘GO:0034968: histone lysine methylation’ were affected (Table S7). In summary, these findings suggest Strip2 plays a vital role in the pluripotency and differentiation of ESCs.

## Discussion

Strip2 was described as a member of the STRIPAK complex, and research so far indicates that it is involved in the regulation of cell growth, proliferation, cell migration and adhesion, neural and vascular development (for review, see [2]) as well as cardiac function [1]. As we previously reported, Strip2 is a nuclear protein involved in the regulation of pluripotency and differentiation in ESCs [3, 4]. Our transcriptome data suggested a potential epigenetic mode of action, thereby enabling Strip2 to regulate differentiation [3, 4]. Hence, we focused on the molecular epigenetic mechanism behind the onset of a normal differentiation process in ESCs regulated by Strip2. To unravel the molecular mechanism, we performed ChIP-Seq, promotor assays, Co-IP and global proteomics on Strip2^−^-ESCs and Strip2^+^ 0-day ESCs and 4-day and 16-day EBs.

Our ChIP-Seq and Co-IP analyses demonstrated that Strip2 interacts with DNA motifs of at least four KRAB-ZFPs including *Rex2* as well as several co-repressor complexes. This finding gives clear evidence that Strip2 may act as a differentiation promotor by interacting with the DNA binding sites of KRAB-ZFPs and/or with proteins which are required to activate the KRAB-ZFP complex. The expression of genes that regulate differentiation is tightly controlled by KRAB-ZFP complexes among other protein complexes. In addition, these proteins complexes selectively interact with transposable elements on the chromatin. Therefore, we also investigated whether Strip2 acts directly via binding to regulatory elements like KRAB-ZFPs or indirectly through protein-protein interactions with the KRAB-ZFP complex, thereby inactivating the effects of ZFPs. The characteristic KRAB domain consists of around 75 amino acids and is found in the N-terminal of about 1/3 of eukaryotic KRAB-ZFPs. KRAB-containing proteins bind to specific DNA sequences via C2H2 (cystein2 histidine2) motifs and normally repress the transcription of several genes, such as the RNA polymerase I, II and III involved in differentiation of ESCs. They can bind to and splice RNA. KRAB-ZFPs are found in the nucleolus and normally act as transcriptional repressors of developmental/differentiation processes by forming the KRAB-ZFP repressor complex [15–17].

Using PLA assays, we could show direct interactions of the Strip2 with the K KRAB-ZFP co-repressor protein complex (NuRD/TRIM28/HDACs/SETDB1), most probably resulting in the methylation and activation of H3K9me3. This hypothesis is supported by the observation that the nucleolar enzymatic activity of Dnmt3 was higher in 4-day and 16-day Strip2^-^-EBs compared to 4-day and 16-day control Strip2^+^-EBs. In this context, it was demonstrated that Dnmt3 methylates H3 to H3K9me3 [18]. It is well established that KRAB-ZFPs bind to transposable elements, which are mobile genomic sequences of DNA, capable of autonomous and non-autonomous duplication. Binding, and therefore repression, of the transposable elements occurs via tandem C2H2 zinc-finger domains and recruits TRIM28 via interactions with the KRAB-A domain [15–17]. TRIM28 stabilizes the binding site of the KRAB-A module and recruits other chromatin-related corepressors, including (1) HDACs members of the of the NuRD subgroup complex (a major protein complex inducing deacetylation of the multiple lysine on histone tails), thereby generating transcriptionally inactive or active chromatin; (2) the heterochromatin protein 1 (HP1; also known as Cbx5), which represses gene expression by converting euchromatin to heterochromatin; and (3) the SET domain bifurcated histone lysine methyltransferase 1 (SETDB1), which specifically methylates histone-H3-Lys-9, thereby generating H3K9me39. The tight interactions of this complex induce a transcriptionally inactive chromatin via methylation of H3 lysine residues to H3K9me3, a process also known as gene silencing by transcriptional repression [15, 17]). It has also been demonstrated that TRIM28 interacts directly with Dnmt3l, which also contributes to the methylation of H3 [17, 19]). In this context, it has been established that repression of transposable elements by KRAB-ZFPs and their co-repressor complex by methylation of H3 to H3K9me3 suppresses developmental processes in vivo [15, 17]. Similarly, it was shown that repression of the transposable elements in ESCs by the KRAB-ZFP complex also regulates the differentiation of ESCs.It has been shown that the KRAB-ZFP42 (also known as Reduced expression 1: Rex1) is a widely used pluripotency marker and is essential to keeping the ESCs in the pluripotent state [20]. It was also reported that in the Strip^-^-ESCs, REX1-depleted human-induced pluripotent stem cells fail to fully differentiate, especially into their mesoderm lineages [20]. ZFP819, a KRAB-ZFP, maintains the ESCs in a pluripotent state by interacting with TRIM28 [21].

The present Strip2^−^-ESCs global proteome findings are consistent with the transcriptome data derived from Strip2^−^-ESCs at different stages of differentiation [3]. It is evident that the level of pluripotent genes remained high during differentiation of the Strip2^−^-ESCs. In addition, during different stages of differentiation of Strip2^−^-ESCs, including the in 16-day Strip2^−^-EBs, somatic cell type specific genes of all lineages were downregulated. Hence, it was implied that Strip2 regulates the initiation of differentiation processes in undifferentiated ESCs toward the primary germ layer cells (ectoderm, mesoderm and endoderm) [3]. In addition, Strip2 depletion in developing zebrafish led to an impaired cardio-myogenesis as well significantly reduced expression levels of the ventricular myosin heavy chain (vmhc) and the cardiac myosin light chain 2 (cmlc2) genes [4]. Our conclusions are summarized in fig. S5 in a form of a graphical abstract.

### Limitations

These findings were derived from mESCs studies and their relevance for differentiation processes of human pluripotent stem cells is of fundamental importance for potential human therapeutic application. Therefore, future studies of Strip2, using human pluripotent stem cells and human cancer cells would be very helpful to evaluate the importance of our studies for human therapeutic application.

## Materials and methods

### Cell culture and differentiation

Mouse ESCs were cultured and differentiated as previously described [22, 23]. Murine CGR8 ESCs from the European Collection of Cell Cultures (ECACC; No. 95011018) were used in this study. The cells were maintained on (0.2%) gelatinized tissue-culture dishes under feeder-free conditions in a standard ESC culture medium, consisting of Glasgow’s minimum essential medium (GMEM; Invitrogen), supplemented with 10% fetal bovine serum (FBS; GIBCO, Thermo Fisher Scientific, Waltham, MA, USA), 2 mM L-glutamine, 100 units/ml leukemia inhibitory factor (LIF-1; Calbiochem), and 50 μM β-mercaptoethanol (β-ME; Invitrogen), as described previously [22, 23]. The cells were passaged on alternate days and maintained confluence between 60 and 70%. ESC differentiation was inducted by the conventional “hanging drop” protocol, as described previously [3]. Briefly, 20 μl hanging drops were made in 10 cm diameter low adhesion dishes from a trypsin-dissociated ESC suspension (2.5 × 10^4^ cells/ml), prepared in differentiation medium (Iscove’s modified Dulbecco’s Medium, IMDM; Life Technologies, Carlsbad, CA, USA), supplemented with 20% fetal calf serum, 1% non-essential amino acids, 2 mM L-glutamine, and 100 μM β-ME. Plates were incubated at 37 °C, under 5% CO_2_ in a humidified incubator for 2 days. EBs that formed were harvested by washing and were resuspended in differentiation medium. The EBs were incubated at 37 °C under 5% CO_2_ under shaking conditions, with a medium change on alternate days.

### Construction of Strip2 active Motif-tag plasmids, chromatin immunoprecipitation and ChIP-sequencing library production

To generate the pAM-1C-Strip2 plasmid, the Strip2 gene was firstly amplified by PCR using the appropriate primers (MG213986, pCMV6-AC-GFP vector; OriGene, Rockvill, MD, USA). The pAM-1C-empty vector was digested with BglII and HindIII and purified by gel extraction. Using homology directed ligation (Takara #638909), the Strip2 was then ligated into linearized the pAM-1C vector. Positive clones of the ESC overexpression Strip2 (referred as Strip2^+^-ESCs) were then confirmed by DNA sequencing analysis. The Strip2^+^-ESCs were transfected using Magnetofection^TM^ transfection technology (OZ Biosciences, France). The pAM-1C-JunD (overexpressing vector) was used as positive control and pAM-1C (empty vector) was used as negative control. Briefly, the transfection cocktail contained 0.5 µl plasmid cDNA (Strip2, JunD and empty vector, respectively) and 1.5 µl of magnetic transfection reagent (called MTX) diluted in 50 µl of serum-free medium. This mixture was transferred to 0.5 µl CombiMag^TM^ reagent (Oz Biosciences) and incubated at room temperature (RT) for 20 min (Supp Fig. 1). This solution was evenly applied on ESCs and MTX. Boost was added according to the manufacturer instructions. The selection of the appropriate clones was initiated immediately after 48 h of transfection by treatment of the cells with 2 µg/ml neomycin to get purified stable transduced overexpressed clones. This process was carried out for five passages. The stable transduced cells were validated for Strip2 expression using qPCR. Expression of eGFP was checked under blue light using a fluorescence microscope (Axiovert 200; Zeiss, Germany).

The ChIP-Seq analysis was carried out using the Active Motif protocol (53022). Briefly, cells were cross-linked with fixation solution for 10 min at 4 °C. Chromatin was then sonicated for 10 cycles (cycle: 30 s ON, 30 s OFF) using Bioruptor (Diagenode, Belgium). 10 µg of AM-Tag antibody (pAb) (Active Motif, 61677) was added to the sheared chromatin and incubated on an end-over-end rotator overnight at 4 °C. Next, 30 μl of protein G agarose beads were added to the ChIP reactions and incubated for an additional 3 h at 4 °C. The chromatin was then de-crosslinked at 55 °C for 30 min and then the temperature was increased to 80 °C for 2 h with proteinase K (Active Motif). ChIP-Seq libraries from ChIP and input DNAs obtained from (Strip2, JunD and empty) were prepared according to the Illumina protocol and sequenced with a 2 × 100 bp or 2 × 74 bp strand-specific protocol on a HiSeq 2500 sequencer (Illumina, San Diego, CA, USA). ChIP-Seq sequencing reads were mapped to the mouse genome (mm10 assembly) using BWA [24]. The resulting BAM files were then analyzed with MACS2 [25] using Strip2: *q* ≤ 0.05; fold-enrichment ≥ 5; Broad Region Calling ON settings in order to identify genomic regions significantly enriched in the investigated proteins in comparison to the total genomic input DNA. For the Strip2-AM tag day 0 ChIP-Seq, three biological replicates were performed, which showed high Pearson Correlation coefficients (0.98), as determined by the BAM-Correlate tool from Deep Tools (bins mode and a bin size of 10 Kb across the whole mouse genome). Motif analyses in active and poised enhancers were performed using the SeqPos motif tool (http://cistrome.dfci.harvard.edu/ap/root) [26] using a width of 600 bp and a *p* value cutoff of 0.001.

### Additional bioinformatic analysis

Functional annotation of Strip2 peaks (i.e., poised, PoiAct, active and primed) was performed with GREAT [27], using the Basal plus extension association rules (Proximal = five Kb upstream, one Kb downstream; Distal = 1000 kb) and the whole mouse genome as background. The top ten most overrepresented terms belonging to various gene annotations (GI Biological Process, GO Molecular Function and InterPro) were identified.

Luciferase assay

Potential interactions of Strip2 with the consensus motif was investigated using the luciferase assay. Briefly, DNA motif associated with Strip2 (GGAGAGAAACCTTACAAATG) was purchased from Integrated DNA Technologies (Coralville, IA USA) and cloned after the SV40 promoter, which controls the expression of luciferase in the pGL3 minimal promoter vector (Promega, Madison, WI, USA), and verified using Sanger sequencing. Luciferase assays were performed in Strip2^+^-ESCs, Strip2^−^-ESCs and transient Strip2 overexpressing ESCs. For this aim, 3 × 10^4^ cells per well were seeded into each well of a 96-well plate. 0.5 µg of each pGL3-based vector was transfected into different ESC cell lines using Lipofectamine 2000 and the medium was changed after overnight incubation. After 48 h, luciferase assays were conducted using the Dual-Glo (Promega) luciferase assay system and results were determined using the renilla luminescence vector as an internal control for all assays. To calculate changes in luciferase activity, driven by the presence of a motif or altered in Strip2 ^−^ -ESCs or in the Strip2 overexpressing ESCs, results were normalized to the empty PGL3 vector without a motif.

### Co-immuno-precipitation of Strip2

To identify the interaction partners of Strip2, we generated an ESC cell line permanently overexpressing Strip2 in undifferentiated wild-type ESCs. Briefly, generation of the Strip2-Turbo-GFP-fusion overexpressing wild-type ESCs (next referred transient Strip2 overexpressing ESCs) was performed by the Magnetofection^TM^ transfection technology using a plasmid with the cDNA cassette encoding for Strip2 and the DNA cassettes for GPF fusion construct and puromycin resistant cassette (Catalogue number: MG213986, pCMV6-AC-GFP; OriGene), as mentioned above. In parallel, a control ESC cell line was generated using a control plasmid without the Strip2 cDNA but with the eGFP and neomycin cassette (next referred as GFP-F + ESCs). Transient Strip2 overexpressing ESCs were passaged for five times under standard cell culture conditions (37 °C, 5% CO_2_). To identify only specific interactions of Strip2 with other proteins, GFP-F + ESCs were used in parallel. Protein extraction and IP was performed using the ChromoTek TurboGFP-Trap Agarose Kit (tbtak-20; Proteintech, Germany), according to the instructions of the kit. The cell extract was prepared using RIPA buffer (Thermo Fisher Scientific). The cell lysate was mixed with TurboGFP-Trap-A beads and equilibrated for 1 h at 4 °C using an end-over-end shaker. After incubation, the samples were washed three times, and then the specific Strip2 bound proteins were eluted using a buffer. The eluted proteins were further analyzed by mass spectrometry.

### Transfection of vectors into undifferentiated ESCs and generation of a constitutive Strip2-silenced ESC line

The shRNA expression vector pGFP-V-RS, targeting mouse Strip2 (TR508344A plasmid) and the scrambled plasmid (TR30013) were purchased from OriGene. The shStrip2 target sequence on its mRNA was 5′ GCAAGACACTAAGGAATGGCTGGAGTTGG 3′, which corresponds to nucleotides 365–393. The scrambled plasmid (TR30013) contains a non-active scrambled sequence cassette (5′ GCACTACCAGAGCTAACTCAGATAGTACT3′). The pGFP-V-RS control, the shRNA vector (TR508344A plasmid), and the scramble shRNA vector (TR30013) were linearized. Generation of the cell lines was performed, as described previously. Briefly, 25 μg of linearized vector was transfected into 10^6^ CGR8 cells, suspended in phosphate-buffered saline (PBS), free of Ca^2+^ and Mg^2+^ salts, using a Bio-Rad Gene Pulser Electroporation System (Bio-Rad, Hercules, CA, USA). The electroporation conditions were 500 μF and 240 V, as described previously. The electroporated cells were cultivated on gelatinized tissue-culture flasks for 2 d and eventually selected for treatment with 2 μg/ml puromycin. On day 10 of selection, green fluorescence was monitored under a blue excitation light through a fluorescence microscope. Afterward, the resistant clones were picked for further experiments and amplified following standard ESCs culture conditions. The clones were passaged at least four times before being used in experiments to attain a stable gene expression profile. The shStrip2 cell line generated with the TR508344A plasmid was used to study gene expression changes during differentiation of mESCs (ESCs and EBs on day 4 and day 16).

### Histone H3 (K9) methyltransferase activity assay

Nuclear extracts were prepared from undifferentiated Strip2^−^-ESCs, 4-day EBs differentiated and 16-day EBs as well as from the appropriate control Strip2^+^-ESCs and the 4-day and 16-day-EBs using Nuclear Extraction Kit (ab113473; Abcam, Cambridge, UK) by following the manufacturer’s protocols. The histone methyltransferase activity of the nuclear fractions of differentiating Strip2^−^-ESCs and differentiating Strip2^+^-ESCs were determined using the histone methyltransferase H3 (K9) activity quantification assay kit (ab113453; Abcam) following the manufacturer’s protocols. Histone H3 acetylation was determined by a histone H3 acetylation assay kit (ab115102; Abcam). The absorbance at 450 nm was measured using a plate reader. Methyltransferase activity was calculated by the formula: activity [OD/h/mg] = (Sample OD – blank OD)/(protein amount [μg] × incubation hour). Histone acetylation activity was calculated by formula: [ng/mg protein] = (Sample OD – blank OD)/(Slope) × 1000.

### Quantitative proteomics applying the “stable isotope labeling by/with amino acids in cell culture” methodology

We performed quantitative proteomics applying the “stable isotope labeling by/with amino acids in cell culture” (SILAC) methodology to identify proteins dysregulated in differentiated Strip2^−^-ESCs in comparison to differentiated Strip2^+^-ESCs. To this aim, we cultured the Strip2^+^-ESCs, with SILAC DMEM containing Arginine and Lysine “heavy” and light isotopes of both amino acids and supplemented with GlutaMAX (1 %, v/v). The “heavy” SILAC DMEM medium consisted of 50 mg of 1-Arg-10 = L-[13C6, 15N4] Arg. HCl, 25 mg of 2-Lys-8 = L-[13C6,15N2] Lys. HCl and 100 mg proline in 500 ml DMEM medium. The composition of the “light” SILAC DMEM medium is similar but the heavy amino acids were replaced with the light (50 mg/500 ml 1-Arg-0 = L-[12C6, 14N4] Arg. HCl, 25 mg/500 ml 2-Lys-0 = L-[12C6,14N2] Lys. HCl). The “heavy” and “light” medium incubated Strip2^−^-ESCs (heavy) and Strip2^+^-ESCs (light) have been cultured for five passages in undifferentiated state before generating the 4-day and 16-day EBs, respectively. The SILAC mass spectroscopy from the isolated undifferentiated Strip2^+^-ESCs and Strip2^−^-ESCs as well as from the Strip2^+^-EBs and Strip2^−^-EBs was analyzed at CECAD/CMMC Proteomics Facility, University of Cologne, in collaboration with Dr. Christian Frese (http://www.cecad.uni-koeln.de/research/core-facilities/proteomics-facility/mass-spectrometers/).

All samples were analyzed on a Q-Exactive Plus (Thermo Fisher Scientific) mass spectrometer that was coupled to an EASY nLC 1000 UPLC (Thermo Fisher Scientific) chromatograph. Peptides were loaded with solvent A (0.1% formic acid in water) onto an in-house packed analytical column (50 cm × 75 µm I.D., filled with 2.7 µm Poroshell EC120 C18; Agilent Technologies, Santa Clara, CA, USA) and chromatographically separated at a constant flow rate of 250 nl/min using 60- or 150 min gradient cycles of 5–30% solvent B (0.1% formic acid in 80 % acetonitrile). The mass spectrometer was operated in data-dependent acquisition mode. The MS1 survey scan was acquired from 300 to 1750 m/z at a resolution of 70,000. The top ten most abundant peptides were isolated within a 2 Da window and subjected to HCD fragmentation at normalized collision energy of 27%. Precursors were dynamically excluded for 20 s.

### Stable isotope labeling by/with amino acids in cell culture data analysis

Bioinformatic analyses of all raw data from mass spectrometry were processed with MaxQuant (version 1.5.3.8; Max Planck Institute of Biochemistry) using default parameters (ref pubmed 27809316). Briefly, MS2 spectra were searched against the Uniprot MOUSE.fasta database, including a list of common contaminants. FDRs on protein and PSM level were estimated by the target-decoy approach to 0.01% (Protein FDR) and 0.01% (PSM FDR), respectively. The minimal peptide length was set to seven amino acids and carbamidomethyolation at cysteine residues was considered as a fixed modification. Oxidation (M) and Acetyl (Protein N-term) were included as variable modifications. For proteome quantification, corresponding SILAC labels were selected. A minimum ratio count of 2 was required. For Co-IP data, the match-between run and label-free quantification options were enabled. For SILAC data, the re-quantify option was enabled. The MaxQuant output was processed as follows: Protein groups flagged as “reverse”, “potential contaminant” or “only identified by site” were removed from the data set. Further analysis was carried out with metascape (Co-IP data) or R and metascape (SILAC data). For SILAC data, proteins with less than four valid values were filtered out. Protein SILAC ratios were log2 transformed and normalized to the median. For every time point, a one sample *t*-test was performed. Proteins with a *p* value < 0.05 and log2 change < −1 or >1 were considered significantly changed and subjected to profile clustering (k-mean cluster analysis). Clusters were generated using the *z*-scored mean value per group. For Co-IP data, proteins with less than two valid values in at least one of the conditions were filtered out. LFQ values were log2 transformed. Missing values were replaced by imputation (width 0.3, down shift 1.9). A two-sample *t*-test was used to determine significantly enriched proteins. Potential contaminants were annotated based on the Crapome repository [28].

### Proximity ligation assay

All PLA reagents were purchased from Duolink (Sigma-Aldrich, St. Louis, MO, USA). PLA was performed according to manufacturer’s instructions. Briefly, differentiating cells from different time point (0-day ESCs, 1-day and 4-day EBs) of Strip2^−^- and Strip2^+^ESCs were fixed with 4% paraformaldehyde, incubated with PBS containing 0.1% Triton X-100, and blocked with Blocking Solution for 60 min. Cells were incubated with primary antibodies (TRIM28 and Strip2, ZFP57 and Strip2) at 4 °C overnight. After washing the cells three times with PBS-T, cells were incubated with oligonucleotide-conjugated secondary antibodies (PLA Probes) PLUS and MINUS for 1 h at 37 °C. Followed by washing with PBS-T and incubated with a ligation mixture (Detection Reagent Red) for 30 min at 37 °C. Cells were washed and incubated with an amplification mixture (Detection Reagent Red) for 100 min at 37 °C. After amplification, cells were washed and rinsed in distilled water. The nuclei were stained with DAPI. Images were captured with an inverted fluorescence microscope (Axiovert 200; Zeiss).

### Statistical analysis

If not otherwise indicated in the text, then analysis was performed using a one-way pairwise ANOVA test or *t*-test and *p* values < 0.05 were considered statistically significant.

## Supplementary information

Supplementary Tables Legends

Supplementary Figure Legends

Figure S1

Figure S2

Figure S3

Figure S4

Figure S5

uncropped original western blot

cdd-author-contribution-form

Table S1

Table S2

Table S3

Table S4

Table S5

Table S6

Table S7

Table S8

Co-authors agreement for co-authorship

## Data Availability

The ChIP-seq raw data were submitted to the National Center for Biotechnology Information (NCBI) under BioProject accession numbers PRJNA856817.
