# Supplementary material for: Epigenetic mechanisms of Strip2 in differentiation of pluripotent stem cells
Source: Cell Death Discov. 2022 Nov 5;8:447. doi: 10.1038/s41420-022-01237-5 (PMC9637104; doi:10.1038/s41420-022-01237-5)
Supplement: Supplementary file 1 — Supplementary Tables Legends [file 41420_2022_1237_MOESM1_ESM.docx]

**Supplementary table legends (tables were provided as excel files)**

**Table S1:** Total number of interaction partner proteins of Strip2 in mESC differentiation (ESCs, 4-day EBs and 16-day EBs) by Q-Exactive Plus.

**Table S2:** Metascape GO/KEGG enrichment analysis (Top 100) of interaction partner proteins of Strip2.

**Table S3:** Metascape enrichment analysis of enriched transcription factor-target interaction networks of interaction partner proteins of Strip2.

**Table S4:** Metascape protein-protein interaction enrichment analysis: total network versus 12 subnetworks.

**Table S5:** Differentially-expressed proteins by Strip2 knockdown in mESC differentiation (ESCs, 4-day and 16-day EBs) by SILAC.

**Table S6:** Protein list from K-means clustering (SILAC).

**Table S7:** Metascape GO/KEGG enrichment analysis of clusters (SILAC).

**Table S8:** Antibody List

**Table S9:** Primer List
