# Supplementary material for: Epigenetic mechanisms of Strip2 in differentiation of pluripotent stem cells
Source: Cell Death Discov. 2022 Nov 5;8:447. doi: 10.1038/s41420-022-01237-5 (PMC9637104; doi:10.1038/s41420-022-01237-5)
Supplement: Supplementary file 2 — Supplementary Figure Legends [file 41420_2022_1237_MOESM2_ESM.docx]

**Supplementary Figures Legends**

**Fig S1.** ChIP-Seq analysis workflow.

**Fig S2**: Generation of transient Strip2 overexpressing ESCs: **A**, Plasmid map and catalogue number (MG213986, pCMV6-AC-GFP; OriGene). **B**, Transfection cocktail contained 0.5µl plasmid cDNA and 1.5 µl of magnetic transfection reagent (called MTX) diluted in 50 µl of serum free medium. This mixture was transferred to 0.5 µl CombiMagTM reagent and incubated at room temperature (RT) for 20 min (figure). This solution was evenly applied on ESCs and MTX. Boost was added according to the manufacturer instructions. The selection of the appropriate clones was initiated immediately after 48 h of transfection by treatment of the cells with 2 µg/ml Neomycin. To get purified stable transduced overexpressed clones. This process was carried out for five passages. **C**, The stable transduced cells were validated for Strip2 expression using qPCR. Expression of the eGFP was checked under blue excitation light using a fluorescence microscope (Axiovert 200; Zeiss). Western blot analysis: Ten µg of the total protein extracts from Strip2 protein expression in the GFP control and Strip2 overexpressed ESCs were separated using 4–12% Bis-Tris Plus precast polyacrylamide gels (Thermo Fisher, Karlsruhe, Germany) by electrophoresis and blotted onto polyvinylidene fluoride membranes (Thermo Fisher, Karlsruhe, Germany). Chemiluminescence detection of Strip2 has been performed using Strip2 polyclonal antibodies (1:250) and GAPDH has been detected using the anti-GAPDH antibody (1:5000) dilutions.

**Fig S3: A**. Representative graphs showing the qRT-PCR gene expression pattern of ChIP-enriched genes (see figure 3A) during differentiation of Strip2^+^ ESCs and Strip2^-^ ESCs (mean ± SD, n=3). **B**, Representative graphs showing the qRT-PCR gene expression pattern of Motif-targeted genes (see figure 3B) during differentiation of Strip2^+^ ESCs and Strip2^-^ ESCs (mean ± SD, n=3).

**Fig S4:** Gene Expression of three embryonic germ-layer markers: Differentiation of transient Strip2 overexpressing ESCs into the three embryonic germ layers using EB formation. **A,** Representative diagrams showing gene expression pattern of Strip2 and pluripotent markers during differentiation of transient Strip2 overexpressing ESCs and Strip2- ESCs (Sox2 and Lin28A, mean ± SD, n=3). **B,** Representative diagrams showing gene expression pattern of ectoderm markers during differentiation of transient Strip2 overexpressing ESCs and Strip2- ESCs (Pax3, MAP2 and ISL-1, mean ± SD, n=3). **C,** Representative diagrams showing gene expression pattern of mesoderm markers during differentiation of transient Strip2 overexpressing ESCs and Strip2- ESCs (Actinin 2, T Brachyury and Troponin-t, mean ± SD, n=3). **D,** Representative diagrams showing gene expression pattern of endoderm markers during differentiation of transient Strip2 overexpressing ESCs and Strip2- ESCs (AFP, Albumin and HNF4A, mean ± SD, n=3).

**Fig S5:** Classic mechanisms of the KRAB-ZFPs for suppression of the transposable elements. M: Methylated Lysine; A: Acetylated Lysine and hypothetical epigenetic action mechanisms of Strip2 supported by several of our findings.
