# Supplementary figures and images for: Epigenetic mechanisms of Strip2 in differentiation of pluripotent stem cells
Source: Cell Death Discov. 2022 Nov 5;8:447. doi: 10.1038/s41420-022-01237-5 (PMC9637104; doi:10.1038/s41420-022-01237-5)

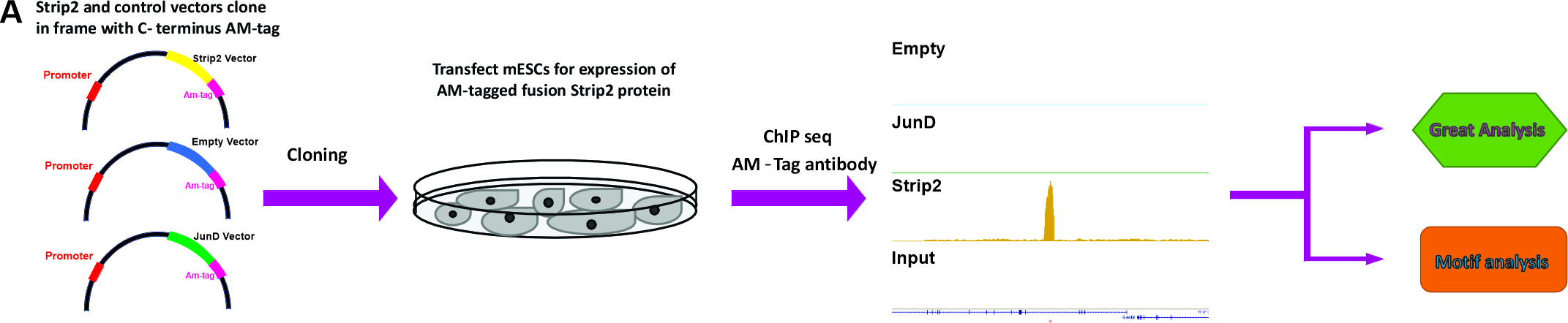

Supplement: Supplementary file 3 — Figure S1 [file 41420_2022_1237_MOESM3_ESM.jpg]

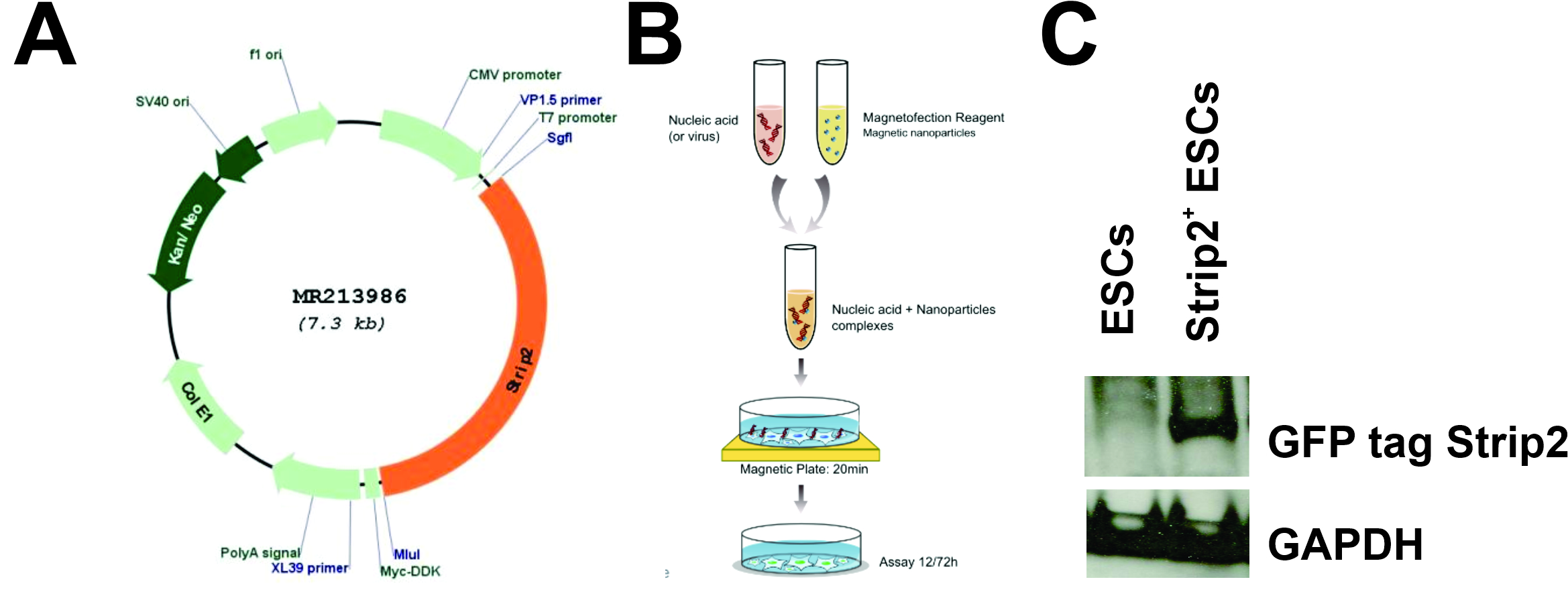

Supplement: Supplementary file 4 — Figure S2 [file 41420_2022_1237_MOESM4_ESM.jpg]

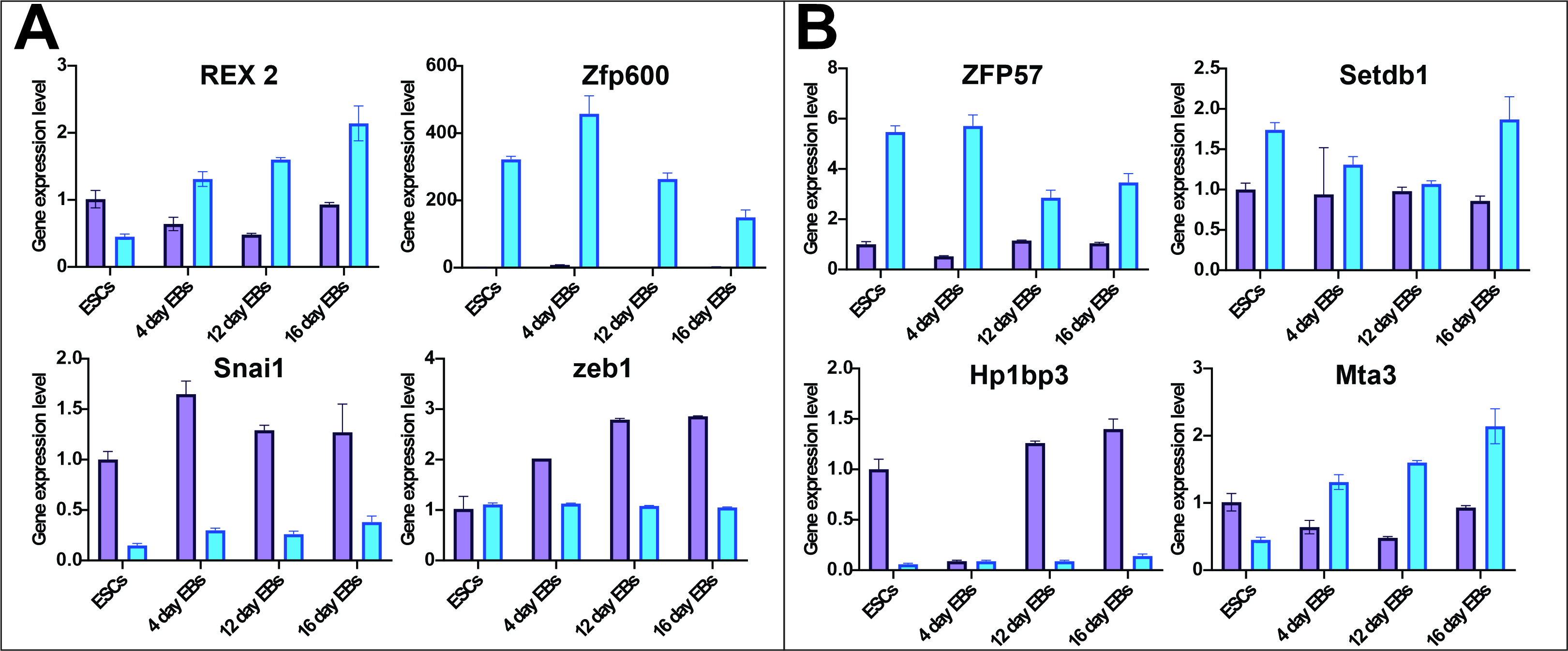

Supplement: Supplementary file 5 — Figure S3 [file 41420_2022_1237_MOESM5_ESM.jpg]

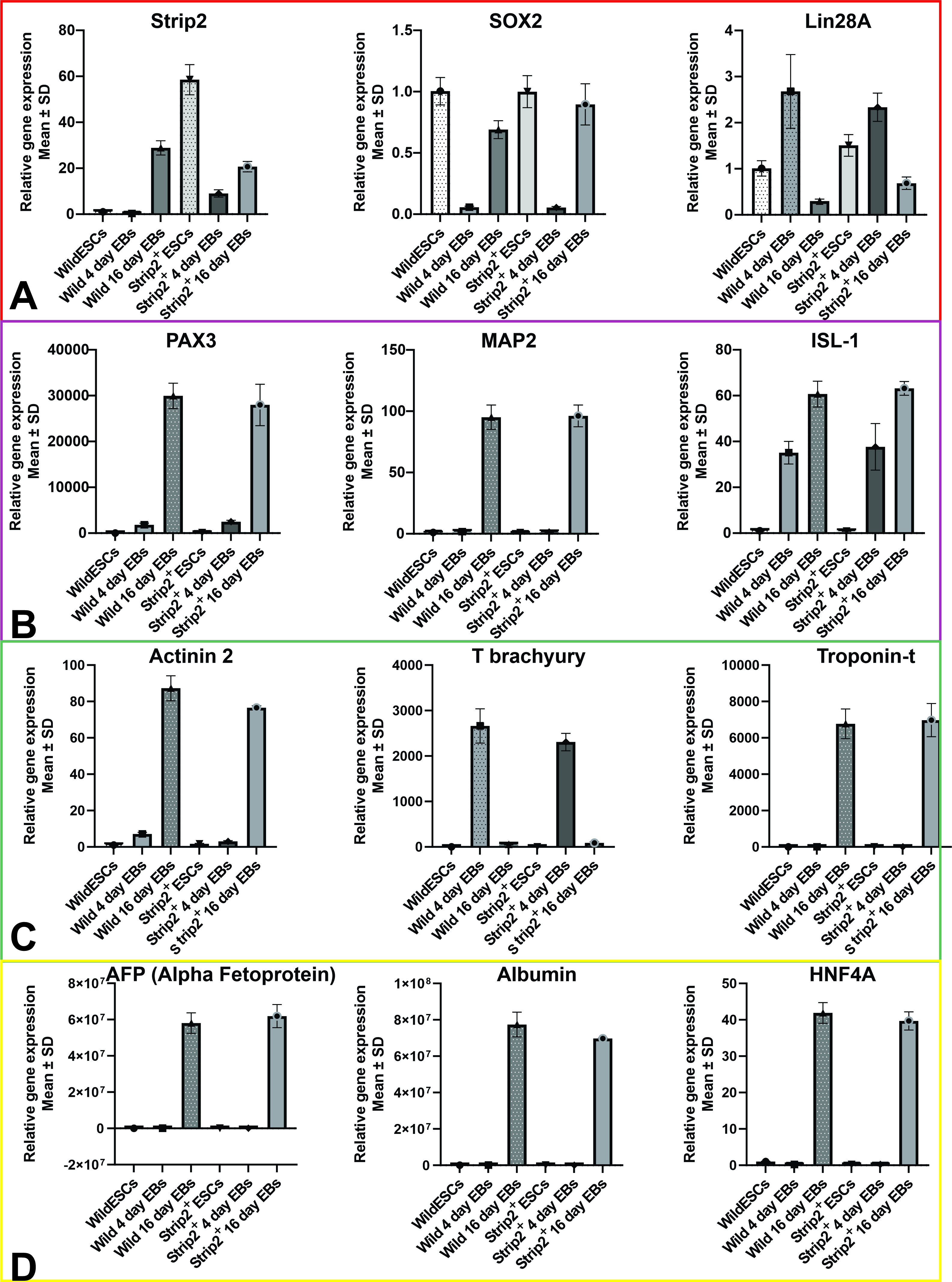

Supplement: Supplementary file 6 — Figure S4 [file 41420_2022_1237_MOESM6_ESM.jpg]

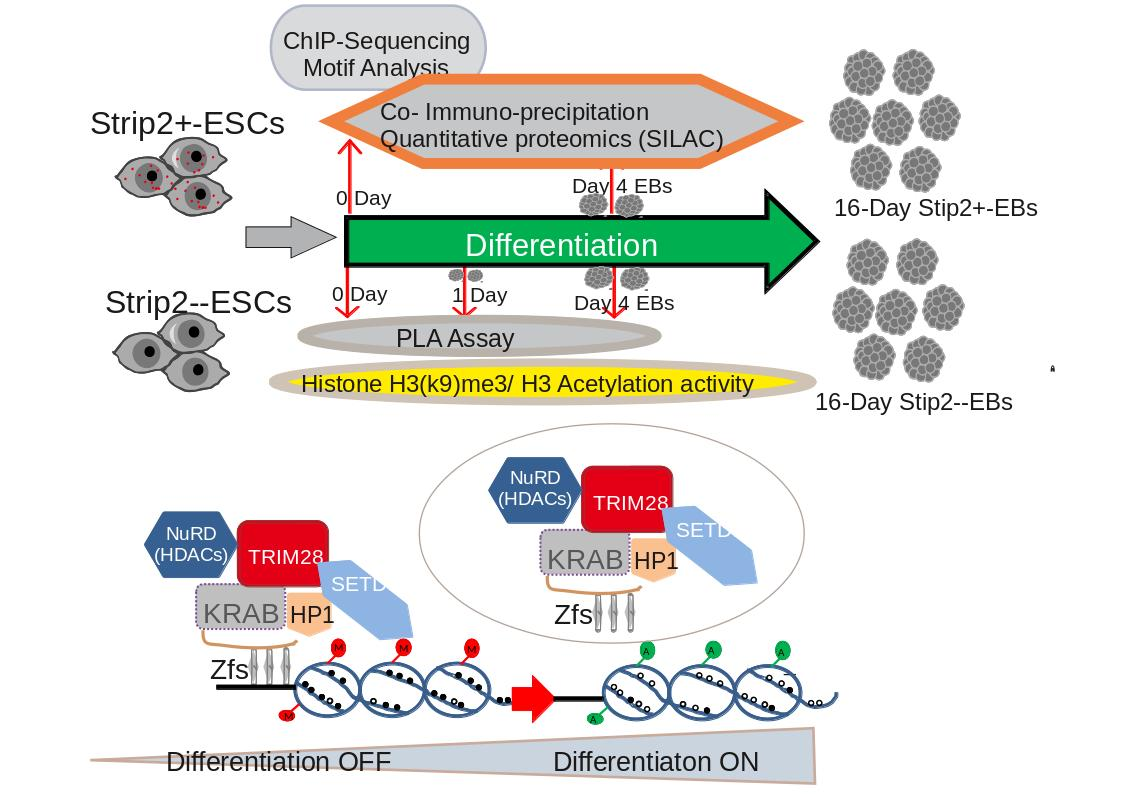

Supplement: Supplementary file 7 — Figure S5 [file 41420_2022_1237_MOESM7_ESM.png]

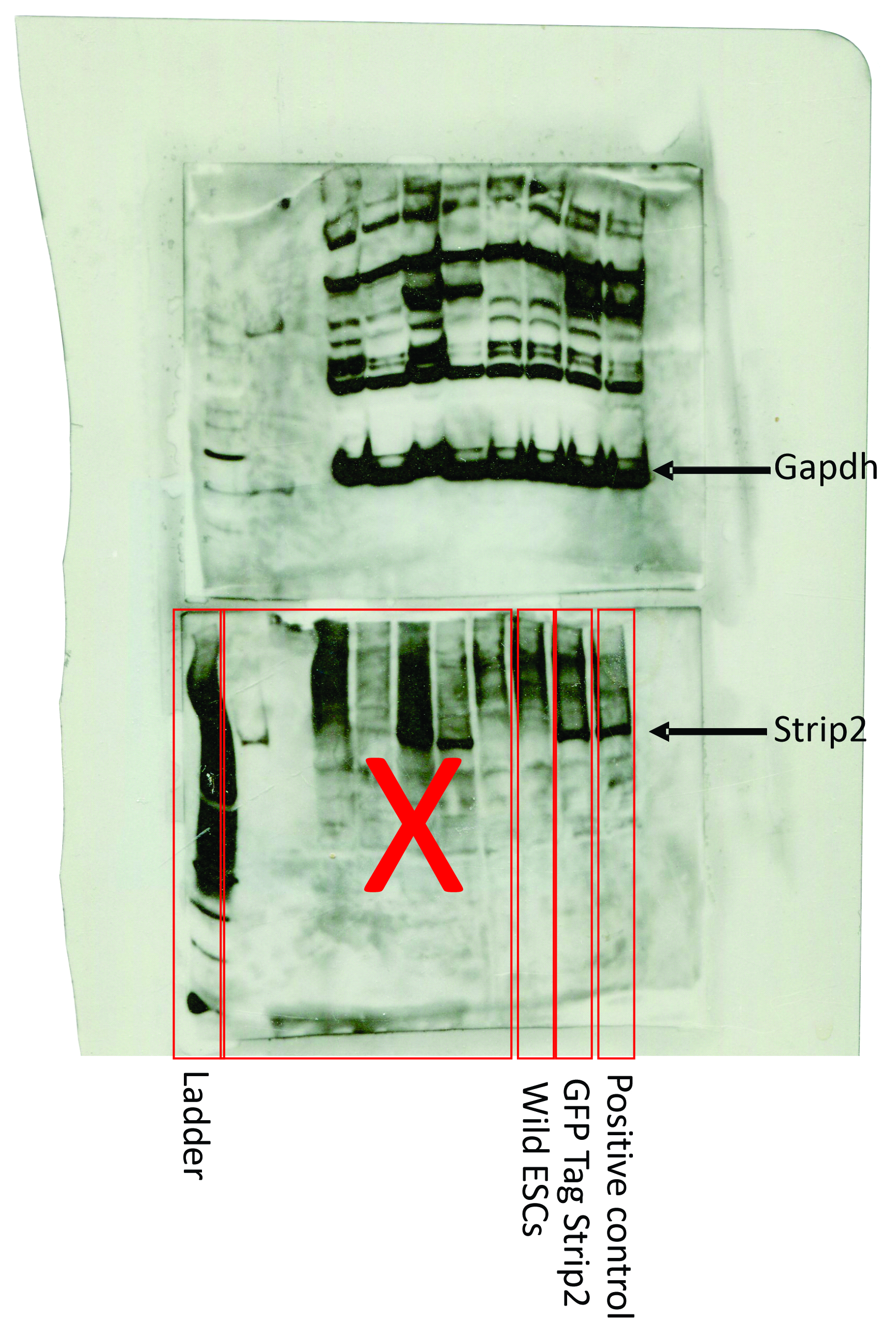

Supplement: Supplementary file 8 — uncropped original western blot [file 41420_2022_1237_MOESM8_ESM.jpg]
