# Supplementary material for: Epigenetic mechanisms of Strip2 in differentiation of pluripotent stem cells
Source: Cell Death Discov. 2022 Nov 5;8:447. doi: 10.1038/s41420-022-01237-5 (PMC9637104; doi:10.1038/s41420-022-01237-5)
Supplement: Supplementary file 9 — cdd-author-contribution-form [file 41420_2022_1237_MOESM9_ESM.pdf]

DECLARATION OF CONTRIBUTIONS TO ARTICLE

ADMC

Manuscript Number: CDDISCOVERY-22-5305R:

Journal Name: *Cell Death Discovery* (the 'Journal')

Proposed Title of the Contribution:

Epigenetic mechanisms of Strip2 in differentiation of pluripotent stem cells (the 'Contribution)

Author(s):

Sureshkumar Perumal Srinivasan , Harshal Nemade, Anna Cherianidou, Luying Peng, Sara Cruz-Molina, Alvaro Rada-Iglesias, Agapios Sachinidis (the 'Authors')

For all *CDD* articles, each person named as an author in the published version must be able to show he or she has contributed substantially to the article.

Authorship credit should be based on 1) substantial contributions to conception and design, acquisition of data, or analysis and interpretation of data; 2) drafting the article or revising it critically for important intellectual content; and 3) final approval of the version to be published. Authors should meet conditions 1, 2 and 3.

Any person who cannot be shown to have made a substantial contribution to the article cannot be listed as an author in the final version. The name of any person who is deemed to have made a minor contribution can, however, appear in the Acknowledgments section of the article.

Please complete the table below to indicate the contributions of all named authors to the manuscript.

Author Full Name:	Specification of Contribution to the Manuscript:
Sureshkumar Perumal Srinivasan	S.P.S. conducted the main experiments, collected all the findings, contributed to the analysis of "SILAC and Co-IP" data and contributed to the writing of the manuscript.
Harshal Nemade	H.N. generated the plasmids for the luciferase assay and pAM-1C-Strip2 plasmid for ChIP.
Anna Cherianidou	A.C. performed the cell culture and PLA assay.
Sara Cruz-Molina	S.C.M. helped in ChIP experiments
Alvaro Rada-Iglesias	Alvaro Rada-Iglesias conducted and supervised the ChiP experiments and contribute to the discussion of the manuscript.
Agapios Sachinidis	A.S. designed and supervised the study, interpreted data, conducted analysis of "Chip" data and wrote the manuscript.
Luying Peng	L.P. contributed to the interpretation of the data.

Please complete the table below to indicate the contributions of all named authors to the figures.

Figure 1:
Sureshkumar Perumal Srinivasan, Agapios Sachinidis, Harshal Nemade, Sara Cruz-Molina & Alvaro Rada-Iglesias

Figure 2:
Sureshkumar Perumal Srinivasan, Agapios Sachinidis, Harshal Nemade & Alvaro Rada-Iglesias

Figure 3:
Sureshkumar Perumal Srinivasan & Agapios Sachinidis,

Figure 4:
Sureshkumar Perumal Srinivasan & Agapios Sachinidis,

Figure 5:
Sureshkumar Perumal Srinivasan, Agapios Sachinidis & Anna Cherianidou

Figure 6:
Sureshkumar Perumal Srinivasan & Agapios Sachinidis,

Signed for and on behalf of the Author(s):	Print Name:	Date:
	Agapios Sachinidis	10-06-2022
