# Supplementary material for: Epigenetic mechanisms of Strip2 in differentiation of pluripotent stem cells
Source: Cell Death Discov. 2022 Nov 5;8:447. doi: 10.1038/s41420-022-01237-5 (PMC9637104; doi:10.1038/s41420-022-01237-5)
Supplement: Supplementary file 18 — Co-authors agreement for co-authorship [file 41420_2022_1237_MOESM18_ESM.pdf]

Agapios Sachinidis

**Von:**	Sureshkumar <sureshkp@uni-koeln.de>
**Gesendet:**	Mittwoch, 19. Oktober 2022 19:50
**An:**	'Agapios Sachinidis'; 'Anna Cherianidou'; alvaro.rada@unican.es; sara.cruz-molina@mpi-muenster.mpg.de; 'hnemade'; luyingpeng@tongji.edu.cn
**Betreff:**	RE: Manuscript: Epigenetic mechanisms of Strip2 in differentiation of pluripotent stem cells in Cell Death Discovery

Dear Prof.,

I agree with the co-authorship of Dr. Peng.

Regards

Suresh

Dr. Sureshkumar
Senior Post Doc
AG Sachinidis
Institut für Neurophysiologie,
Medizinische Fakultät,
Klinikum der Universität zu Köln,
Robert-Koch Str,39,KÖLN,D-50931
Phone: +49-221-478-6978

From: Agapios Sachinidis <a.sachinidis@uni-koeln.de>
Sent: 19 October 2022 15:32
To: sureshkp <sureshkp@uni-koeln.de>; Anna Cherianidou <acherian@uni-koeln.de>; alvaro.rada@unican.es; sara.cruz-molina@mpi-muenster.mpg.de; hnemade <hnemade@uni-koeln.de>; luyingpeng@tongji.edu.cn
Subject: Manuscript: Epigenetic mechanisms of Strip2 in differentiation of pluripotent stem cells in Cell Death Discovery

Dear co-authors,
as you probably know our manuscript in Cell Death Discovery is accepted for publication. Since Dr Luying Peng helped me to interpret the data I put him as a co-author. The journal asked me now that you also agree that Dr, Peng will be also a co-author in our manuscript. To avoid any delay, please reply to my email as soon as possible confirming that you agree with the co-authorship of Dr Peng.

Best regards
Agapios

Prof. Agapios Sachinidis (PhD)
University of Cologne
Institute of Neurophysiology and
Center for Molecular Medicine Cologne (CMMC)
Robert-Koch-Str. 39
50931 Cologne
Germany
Tel: +49 (0) 221 478 7373
Fax: +49 (0) 221 478 6965

Agapios Sachinidis

**Von:**	Sara De La Cruz Molina <sarade@miltenyi.com>
**Gesendet:**	Donnerstag, 20. Oktober 2022 13:05
**An:**	Agapios Sachinidis
**Betreff:**	manuscript

Dear Prof. Sachinidis,

Thanks for your Email and for including me in this manuscript.

Since I am still a guest Scientist at the MPI Münster, I think you can put this as my affiliation:

Max Planck Institute for Molecular Biomedicine, Muenster, Germany

I agree on having Dr. Luying Peng as co-author.

Best regards,

Sara

From: Agapios Sachinidis <a.sachinidis@uni-koeln.de>
Sent: Donnerstag, 20. Oktober 2022 10:15
To: Sara De La Cruz Molina <sarade@miltenyi.com>
Subject: [EXTERNAL]

WARNING: This email was sent from an address outside the company. Do not click on any links or open any file attachments unless you recognize the sender and are confident the content is safe.

Dear Sara,
I sent you the mail for your agreement that you agree that Dr Luying Peng become as a co-author.

Once again, thank you so much for your impact on this publication. Please check if your affiliation would be ok as it stands in the manuscript.

Best
Agapios
Prof. Agapios Sachinidis (PhD)
University of Cologne
Institute of Neurophysiology and
Center for Molecular Medicine Cologne (CMMC)
Robert-Koch-Str. 39
50931 Cologne
Germany
Tel: +49 (0) 221 478 7373

Agapios Sachinidis

Von: hnemade@uni-koeln.de
Gesendet: Mittwoch, 19. Oktober 2022 15:37
An: Agapios Sachinidis
Cc: sureshkp; Anna Cherianidou; alvaro.rada@unican.es; sara.cruz-molina@mpi-muenster.mpg.de; luyingpeng@tongji.edu.cn
Betreff: Re: Manuscript: Epigenetic mechanisms of Strip2 in differentiation of pluripotent stem cells in Cell Death Discovery

Dear Prof.,

'I agree with the co-authorship of Dr. Peng.'

regards,
Harshal.

Quoting Agapios Sachinidis <a.sachinidis@uni-koeln.de>:

> Dear co-authors,
>
> as you probably know our manuscript in Cell Death Discovery is
> accepted for publication. Since Dr Luying Peng helped me to interpret
> the data I put him as a co-author. The journal asked me now that you
> also agree that Dr, Peng will be also a co-author in our manuscript.
> To avoid any delay, please reply to my email as soon as possible
> confirming that you agree with the co-authorship of Dr Peng.
>
>
>
> Best regards
>
> Agapios
>
>
>
> Prof. Agapios Sachinidis (PhD)
>
> University of Cologne
>
> Institute of Neurophysiology and
>
> Center for Molecular Medicine Cologne (CMMC)
>
> Robert-Koch-Str. 39
>
> 50931 Cologne
>
> Germany

Agapios Sachinidis

**Von:**	acherian <acherian@uni-koeln.de>
**Gesendet:**	Mittwoch, 19. Oktober 2022 20:19
**An:**	Sureshkumar; 'Agapios Sachinidis'; alvaro.rada@unican.es; sara.cruz-molina@mpi-muenster.mpg.de; 'hnemade'; luyingpeng@tongji.edu.cn
**Betreff:**	RE: Manuscript: Epigenetic mechanisms of Strip2 in differentiation of pluripotent stem cells in Cell Death Discovery

Dear Professor,

I agree with the co-authorship of Dr.Peng.

Kind regards,
Anna

-------- Original message --------
From: Sureshkumar <sureshkp@uni-koeln.de>
Date: 19/10/2022 19:49 (GMT+01:00)
To: 'Agapios Sachinidis' <a.sachinidis@uni-koeln.de>, 'Anna Cherianidou' <acherian@uni-koeln.de>, alvaro.rada@unican.es, sara.cruz-molina@mpi-muenster.mpg.de, 'hnemade' <hnemade@uni-koeln.de>, luyingpeng@tongji.edu.cn
Subject: RE: Manuscript: Epigenetic mechanisms of Strip2 in differentiation of pluripotent stem cells in Cell Death Discovery

Dear Prof.,

I agree with the co-authorship of Dr. Peng.

Regards

Suresh

Dr. Sureshkumar

Senior Post Doc

AG Sachinidis

Institut für Neurophysiologie,

Agapios Sachinidis

**Von:**	Rada Iglesias, Alvaro <alvaro.rada@unican.es>
**Gesendet:**	Mittwoch, 19. Oktober 2022 15:36
**An:**	Agapios Sachinidis; sureshkp; Anna Cherianidou; sara.cruz-molina@mpi-muenster.mpg.de; hnemade; luyingpeng@tongji.edu.cn
**Betreff:**	Re: Manuscript: Epigenetic mechanisms of Strip2 in differentiation of pluripotent stem cells in Cell Death Discovery

Dear Agapios,

First of all, congratulations on the paper acceptance. I confirm that I agree with the co-authorship of Dr.Peng.

Best regards,

Álvaro

Álvaro Rada-Iglesias
Científico Titular/Tenured Scientist - CSIC
Transcriptional regulation in development and congenital disease
Institute of Biomedicine and Biotechnology of Cantabria (IBBTEC), CSIC/University of Cantabria
Phone: (+34) 942 203932
Email: alvaro.rada@unican.es
Mailing address: IBBTEC. C/ Albert Einstein 22, PCTCAN, 39011 Santander

---

From: Agapios Sachinidis <a.sachinidis@uni-koeln.de>
Sent: Wednesday, October 19, 2022 3:32 PM
To: sureshkp <sureshkp@uni-koeln.de>; Anna Cherianidou <acherian@uni-koeln.de>; Rada Iglesias, Alvaro <alvaro.rada@unican.es>; sara.cruz-molina@mpi-muenster.mpg.de <sara.cruz-molina@mpi-muenster.mpg.de>; hnemade <hnemade@uni-koeln.de>; luyingpeng@tongji.edu.cn <luyingpeng@tongji.edu.cn>
Subject: Manuscript: Epigenetic mechanisms of Strip2 in differentiation of pluripotent stem cells in Cell Death Discovery

Dear co-authors,
as you probably know our manuscript in Cell Death Discovery is accepted for publication. Since Dr Luying Peng helped me to interpret the data I put him as a co-author. The journal asked me now that you also agree that Dr, Peng will be also a co-author in our manuscript. To avoid any delay, please reply to my email as soon as possible confirming that you agree with the co-authorship of Dr Peng.

Best regards
Agapios

Prof. Agapios Sachinidis (PhD)
University of Cologne
Institute of Neurophysiology and
Center for Molecular Medicine Cologne (CMMC)
Robert-Koch-Str. 39
50931 Cologne
